# Developing guidelines for the translation and cultural adaptation of the Montreal Cognitive Assessment: scoping review and qualitative synthesis

**DOI:** 10.1192/bjo.2021.1067

**Published:** 2022-01-07

**Authors:** Ghazn Khan, Nadine Mirza, Waquas Waheed

**Affiliations:** Centre for Primary Care and Health Services Research, The University of Manchester, UK; Centre for Primary Care and Health Services Research, The University of Manchester, UK; Centre for Primary Care and Health Services Research, The University of Manchester, UK.

**Keywords:** Cross-cultural, dementia, ethnic minority, psychometrics, transcultural psychiatry

## Abstract

**Background:**

Ethnic minorities in countries such as the UK are at increased risk of dementia or minor cognitive impairment. Despite this, cognitive tests used to provide a timely diagnosis for these conditions demonstrate performance bias in these groups, because of cultural context. They require adaptation that accounts for language and culture beyond translation. The Montreal Cognitive Assessment (MoCA) is one such test that has been adapted for multiple cultures.

**Aims:**

We followed previously used methodology for culturally adapting cognitive tests to develop guidelines for translating and culturally adapting the MoCA.

**Method:**

We conducted a scoping review of publications on different versions of the MoCA. We extracted their translation and cultural adaptation procedures. We also distributed questionnaires to adaptors of the MoCA for data on the procedures they undertook to culturally adapt their respective versions.

**Results:**

Our scoping review found 52 publications and highlighted seven steps for translating the MoCA. We received 17 responses from adaptors on their cultural adaptation procedures, with rationale justifying them. We combined data from the scoping review and the adaptors’ feedback to form the guidelines that state how each question of the MoCA has been previously adapted for different cultural contexts and the reasoning behind it.

**Conclusions:**

This paper details our development of cultural adaptation guidelines for the MoCA that future adaptors can use to adapt the MoCA for their own languages or cultures. It also replicates methods previously used and demonstrates how these methods can be used for the cultural adaptation of other cognitive tests.

## Dementia and minor cognitive impairment prevalence

Globally, 46.8 million people live with dementia, and this is expected to increase to 115.4 million by 2050.^1,2^ Minor cognitive impairment (MCI), which may later develop into dementia, is also prevalent in up to 42% of the global population over 60 years of age.^3^

Within the UK alone, there are over 850 000 people with dementia and it has become the leading cause of death.^4,5^ By 2025, this number is expected to rise to 1 million.^1^ MCI is also estimated in between 5 and 20% of people over 65 years of age in the UK,^6^ with one in ten having a chance of developing further dementia.^7^

## Dementia and MCI in minority populations

Because of the impact of globalisation, many Western countries, including the UK, have formed significant ethnic minority populations; currently 14% of the UK population identify as an ethnic minority,^8^ and this is estimated to increase to 20% by 2051.^9^ This includes non-English speakers. Currently, there are an estimated 88 languages other than English spoken in the UK, with over 8% of the population not having English as a first language, and over 864 000 people struggling or unable to speak it.^8^

In countries such as the UK, there is also a growing ageing ethnic minority population.^10^ Whereas previously many ethnic minorities would return to their home country as they grew older, many people from minority backgrounds who migrated in between the 1950s and 1970s as young adults are now choosing to permanently reside in the UK in their old age.^11^

These older ethnic minorities are at particularly high risk for receiving a diagnosis of dementia.^10,12^ They are highly susceptible to risk factors known to increase the likelihood of dementia, showing higher prevalence for diseases and conditions such as diabetes, heart disease, hypertension and obesity.^13–15^ Furthermore, ethnic minorities are more likely to come from low socioeconomic backgrounds, which is associated with their likelihood of developing dementia.^16,17^

## Cognitive testing in ethnic minorities

For an early and accurate diagnosis of dementia, the use of cognitive tests in the diagnostic process is vital.^18^ However, a key limitation of cognitive tests available to us is that they are designed for people from European cultures, fluent in the English language.^19,20^

Therefore, when administering cognitive tests to ethnic minorities, there is reduced sensitivity and specificity; we see higher instances of false positive scores (where dementia or MCI is incorrectly detected by the test in a cognitively healthy person) and false negative scores (where a person's dementia or MCI is not detected by the test) within ethnic minorities compared with their English-speaking European counterparts.^21–24^

To overcome this issues, cognitive tests are often translated into target languages. However, translation alone does not address these increased rates of false positive and false negative scores that may also arise from cultural differences. Ethnic minorities may not be familiar with Western concepts such as questions about the Western calendar, Western names or Western historical and general knowledge, which are used for assessing cognitive domains such as orientation, language and memory tasks.^23^ Therefore, when assessing for dementia, cultural bias can significantly affect the validity of these cognitive tests.^25,26^

Flaherty et al^27^ explored this in their research on the five dimensions of cross-cultural equivalence that can be used to minimise cultural bias and measurement errors in cross-cultural testing.^27^ These dimensions are content, conceptual, criterion, semantic and technical equivalence, and should be maintained by tests when they are adapted for different language and culture backgrounds.^27^

In the context of cognitive tests for dementia and MCI technical equivalence (how the test is administered is culturally appropriate for the person being assessed, e.g. language the test is administered in) and semantic equivalence (once translated, the meanings of the test questions are the same as the original test questions, e.g. accurate translation of questions) are retained through simply translating the cognitive test into a target language.^27^

However, without accounting for cultural differences as well, there is still a loss of three of the five dimensions. These are content equivalence (the questions of the test are culturally relevant to the cultural context of the person being assessed), criterion equivalence (after accounting for culture, the test as a whole still assesses for dementia and MCI) and conceptual equivalence (after accounting for culture, the individual test questions still accurately assess the cognitive domains they were meant to at the original level of difficulty).^27^

To ensure that all five dimensions of cross-cultural equivalence are maintained, solutions such as altering cut-off scores of cognitive tests for different ethnic minorities and non-English-speaking groups has been suggested.^28,29^ However, this has still been shown to reduce the sensitivity and specificity.^28,29^ With developing and piloting new cognitive tests not being the most feasible solution, adapting existing cognitive tests for different languages and cultures has been proposed.^25,26,30^

## The Montreal Cognitive Assessment

The Montreal Cognitive Assessment (MoCA) is a cognitive test, developed to screen and assess for dementia and MCI.^31^ This tool was developed by Nasreddine et al,^31^ and is currently used to aid physicians primarily in detecting MCI, a state that can progress to dementia. The test lasts 10 minutes and is given a score out of 30. There are 13 questions that assess the cognitive domains attention, fluency, memory, language, orientation, visuospatial abilities and executive functioning (see Table 1).

**Table 1:** Questions of the Montreal Cognitive Assessment

Question number	Task/question
1. Executive functioning – alternate trail making	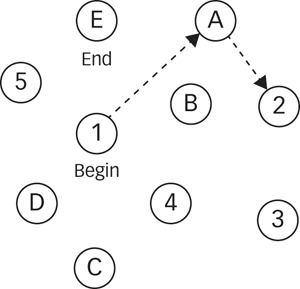 Join the numbers and the letters in the order they ascend.
2. Visuospatial abilities – cube task	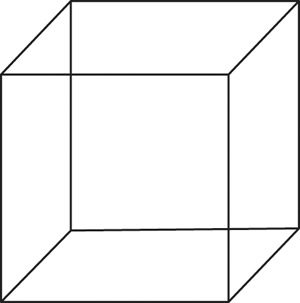 Copy the cube.
3. Visuospatial abilities – clock task	Draw a clock with the time showing ten past eleven.
4. Language – naming	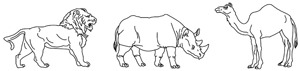 Name each of the three images.
5. Memory – anterograde	Repeat and remember the words face, velvet, church, daisy and red.
6. Attention – digit span	Read one list of digits in a forward order and another list of digits in a backword order.
7. Attention – vigilance	Read a list of letters and tap your hand each time the letter A appears.
8. Attention – serial 7s	Take away 7 from 100 and keep taking 7 away from the new number for five trials (serial 7s).
9. Language – repetition	Repeat the sentences ‘I only know that John is the one to help today’ and ‘The cat always hid under the couch when the dogs were in the room’.
10. Language – fluency	List as many words as you can starting with the letter F in 1 minute.
11. Abstraction	Identify the similarity between ‘a train and a bicycle’ and ‘a watch and a ruler’.
12. Memory – recall	Repeat the word remembered in Question 5. Memory – anterograde.
13. Attention – orientation	Answer what date, month, year, and day, it is and which place and city.

A validation study conducted in 2005 found that the MoCA is a better substitute for the assessment of MCI and dementia than the Mini-Mental State Examination (MMSE).^31^ Since its inception, the MoCA has been used to assess for several variations of dementia and associated diseases such as Parkinson's disease, vascular dementia and Huntington's disease.^32–34^

A recent systematic review of cross-cultural applicability of the MoCA investigated the effect language and cultural diversity have on assessing for MCI, using this cognitive screening tool.^35^ The review reported a wide range of cut-off scores cross-culturally (both across countries and within regions), making it difficult to identify cut-off scores to differentiate between cognitively healthy individuals and those with MCI, as scores could be considered either cognitively healthy or indicative of MCI depending on which cut-offs are used. Furthermore, the review indicates very few studies having conducted validation studies on their adapted measures, meaning sensitivity and high false positive rates. Thus, the review highlights how it can be difficult to identify recommended adjusted cut-offs according to culture, age and education level.^35^

Therefore, as suggested, culturally adapting the existing MoCA would be the best approach to make it suitable for different languages and cultural contexts. In fact, the MoCA is currently widespread in use across over 50 different language and cultural versions.^35^ Yet, despite there being literature present that highlights the various adaptations of the MoCA and their development, using methods such as the use of global guidelines or the involvement of experts, there is no consensus on which questions must be culturally adapted and why.^36,37^ There is also a lack of standard steps and procedures that can be undertaken to translate and culturally adapt the MoCA.

## Culturally adapting cognitive tests

Waheed et al^38^ proposed that every cognitive test should have its own set of specific guidelines on culturally adapting each of its questions. These would be developed through an incorporation of previous literature and feedback from those who have previously adapted it.^38^

At present, there are only one set of guidelines that have been developed for the cultural adaptation of a specific cognitive test.^38^ These are for the Addenbrooke's Cognitive Examination Version III (ACE-III), a gold standard for the diagnostic accuracy of cognitive impairment and dementia.^39,40^ These guidelines were developed through a multi-stage qualitative study combining findings from a systematic review on the translation and cultural adaptations of the ACE-III with feedback received via questionnaires from previous adaptors of the ACE-III.^36,38,41^

The findings identified how adaptors of the ACE-III had adapted each test question for their culture, and their rationale for how they applied changes to test questions based on cultural differences. Through these findings, steps were identified for the cultural adaptation of each ACE-III test question, and these were formatted into a set of guidelines that stated which questions of the ACE-III required cultural adaptation and how they had been adapted, with justification for the adaptation process.^38^ As a demonstration of their applicability, these guidelines were then implemented, with input from focus groups, to develop an Urdu version of the ACE-III.^38^

This Urdu version of the ACE-III was then culturally validated by administering it to 25 cognitively health Urdu speakers, who were then interviewed to determine if the test questions matched their language and cultural context.^41^ Waheed et al^38^ determined that when translating and culturally adapting a test, particularly cognitive tests that are heavily reliant on language and culture, developing cultural adaptation guidelines for the test, implementing them to create a culturally appropriate version of the test and culturally validating the test are all steps that should precede a psychometric validation of the new version of the test.^38^

## Aims

In this paper, we will describe undertaking the initial portion of this methodology to develop guidelines, this time specifically for translating and culturally adapting the MoCA. Because of the widespread availability of its existing adapted versions, it was a suitable cognitive test to develop guidelines for. The research team also consisted of two of the original developers of the ACE-III guidelines (N.M. and W.W.).

We developed the guidelines for culturally adapting the MoCA through a scoping review of the literature, using a systematic search strategy and utilising feedback from those who had adapted the MoCA for their respective cultures and languages. By developing these guidelines, we endeavoured to provide a series of evidence-based instructions that future adaptors of the MoCA can incorporate into their culturally adapted protocols, with evidence to allow for the maintaining of content, criterion and conceptual equivalence.^27^

## Method

A multi-stage qualitative approach was undertaken to develop guidelines for translating and culturally adapting the MoCA. Step 1 was a scoping review of publications on translated and culturally adapted versions of the MoCA. Step 2 involved collecting feedback from previous MoCA adaptors. Finally, in step 3, data was collated from steps 1 and 2 to form the guidelines.

It is important to note that steps 1 and 2 are mutually exclusive, and can be conducted independently. However, both steps must be complete to allow for step 3, the collation of their findings. This research took place at the Centre for Primary Care and Health Services Research, The University of Manchester.

### Step 1: scoping review

We conducted a scoping review with a systematic search of all primary publications of translations and cultural adaptations of the MoCA. The methods of this review followed a similar systematic review of the ACE-III conducted by Mirza et al.^36^

#### Search strategy

Because of the nature of the review, the search was conducted with healthcare-based electronic databases EMBASE, Medline and PsycINFO. The search terms were ‘Montreal cognitive assessment OR MoCA’ AND ‘dementia’ OR ‘Alzheimer*’ OR ‘cognitive’. A broad search was conducted to yield the maximum number of papers. Furthermore, a filter was applied to restrict the search period from April 2005 (as that was when the first paper on the MoCA was published) to July 2020.^31^ In addition, we utilised Web of Science and Scopus to see if there were any publications that cited the original paper by Nasreddine et al.^31^

#### Inclusion and exclusion criteria

Papers that mentioned a translated and/or culturally adapted version of the MoCA and were the original paper to have reported that adaptation were included. This included but were not limited to validation studies.

Papers that did not mention a translated or culturally adapted version of the MoCA were excluded.

#### Study selection

The results of the searches in each database were exported to Endnote (Endnote Basic for Clarivate, The Endnote Team, Philadelphia, USA; see https://endnote.com/downloads) and duplicates were removed. The selection of studies was conducted in two steps. First, the titles and abstracts were screened, and following this, the full texts of selected papers were accessed and compared against the eligibility criteria.

When the full texts were unavailable, the authors of the publications were contacted to provide additional information. A final attempt to obtain the publications was made by contacting the respective journals and putting in a request.

In addition, when requesting data from adaptors of the MoCA in step 2, we enquired whether their adapted MoCAs were mentioned in any earlier publications, and as a result, we were able to attain further sources.

#### Data extraction

The following data was extracted from the full text versions of the selected papers: authors, year of publication, country and language the MoCA was adapted for, and any text that described how they translated and adapted test questions for cultural differences (translation and cultural adaptation procedures).

The reported translation procedures of each paper were broken down into mutually exclusive steps. The translation steps of all papers were then merged, and duplicates were removed, to create a list of all potential steps on translating the MoCA.

The reported cultural adaptation procedures of each paper were reviewed to identify which questions of the MoCA had been culturally adapted and how. The cultural adaptation procedures for each question across papers were merged and duplicates were removed. This created a list of mutually exclusive steps for each question of the MoCA on how to culturally adapt it, along with the accompanying rationale.

#### Reporting of the cultural adaptation and translation procedures, and quality assessment

**Fig. 1:**
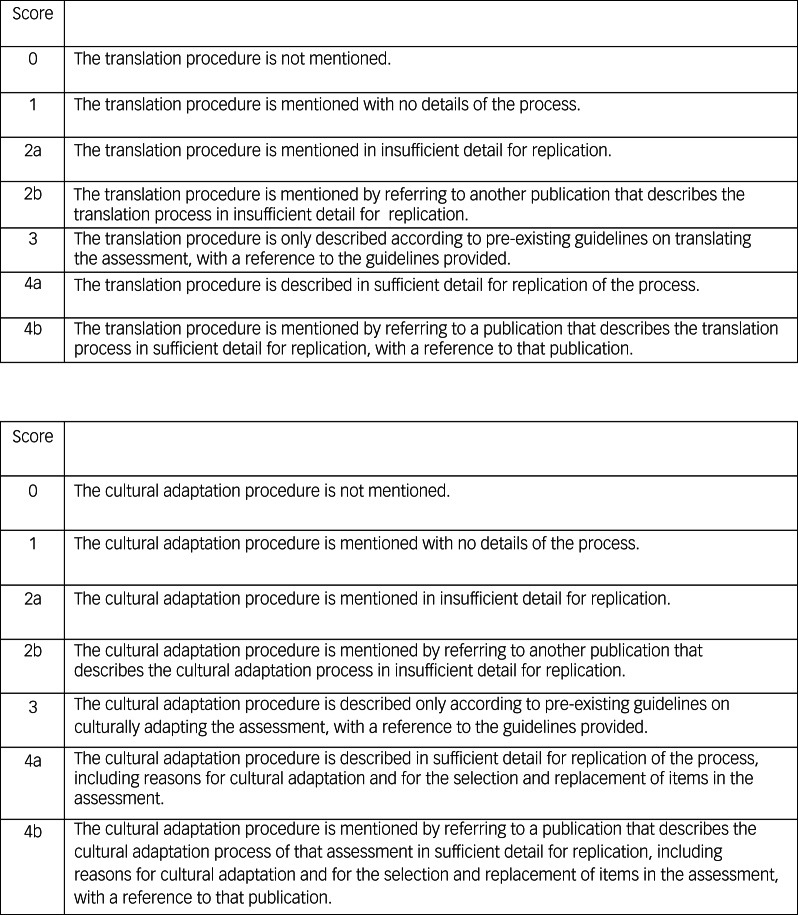
The Manchester Translation Reporting Questionnaire and the Manchester Cultural Adaptation Reporting Questionnaire.

Two authors (G.K. and N.M.) assessed the quality of the reported translation and cultural adaptation procedures of the papers by using the Manchester Translation Reporting Questionnaire (MTRQ) and the Manchester Cultural Adaptation Reporting Questionnaire (MCAR) (see Fig. 1). These scales were initially used to assess the reported adaptations procedures of publications on the ACE-III by Mirza et al.^36,38^ These scales help display which papers reported their translation and cultural adaptation procedures in sufficient detail to be replicated by future adaptors.

Each questionnaire is a seven-point rating scale that quantifies the quality of the reporting of the translation and cultural adaptation of a health measure, particularly cognitive tests. A score of 0 means that translation or cultural adaptation was not mentioned; 1 means that it was mentioned but with no details of the process; 2a or 2b means that a description of translation or cultural adaptation was mentioned either in the paper or referring to another paper, respectively, but the description was in insufficient detail for replication; 3 means that the translation or cultural adaptation described according to pre-existing guidelines, with a reference provided; and 4a or 4b means that a description of translation or cultural adaptation was mentioned either in the paper or referring to another paper, respectively, and the description was in sufficient detail for replication.

It is important to note that the MTRQ and MCAR do not assess the quality of the actual translation and cultural adaptation, as papers may not always report in detail what procedures they undertook. Instead, rating is based on the extent to which translation and cultural adaptation can be replicated from the information reported.

### Step 2: feedback from previous MoCA adaptors

We aimed to gather a description of the cultural adaptation procedures and associated rationale from previous adaptors of the MoCA, who had translated and culturally adapted it for their respective language and culture.

#### Participants

Previous adaptors of the MoCA were identified as those who had made their version available on the MoCA website.^42^

#### Materials

We downloaded all adaptations of the MoCA from the website, which currently hosts the MoCA and all its versions, currently providing 62 translations and cultural adaptations of the test, across 54 languages. This included one English version for Singapore and six Chinese versions, highlighting the impact of culture beyond language differences. Using the online translation application Google Translate (Google Translate for Google, Google, California, USA; see https://translate.google.co.uk/), we translated the MoCA versions to English.^43^ We checked which questions had been directly translated and which questions had been culturally adapted beyond a change in language.

We developed a uniform set of questionnaires for each version of the MoCA that had culturally adapted questions. These were based on the questionnaires used to develop guidelines for culturally adapting the ACE-III.^38^

These questionnaires asked participants to first mention if their version of the MoCA was mentioned in any publications, and to list them. Following this, it presented them with the MoCA questions they had culturally adapted along with the question's original counterpart. For each of these questions, it enquired why cultural changes were needed, and the rationale behind how they changed it from the original to their version (see Supplementary Appendix 1 available at https://doi.org/10.1192/bjo.2021.1067 for a sample questionnaire).

Questionnaires for 45 of the 62 versions were developed (72.6%). For the remaining 17 versions, we could not make questionnaires as we were unable to create back translations of the test in English. This was because of a lack of resources in terms of electronic translation applications and translators available. These were the Bengali, Kannada, Lithuanian, Malayalam, Marathi, Myanmar, Persian, Russian, Sinhalese, Slovak, Slovenian, Swahili, Tamil, Telugu, Thai, Ukrainian and Vietnamese versions. Therefore, they have been excluded from the analysis (27.4%).

#### Procedure

Contact information for the adaptors of the MoCA was tracked down by first checking the MoCA version, followed by contacting the MoCA website. If a name was identified, we searched PubMed, ResearchGate and LinkedIn for an active email address. We also searched respective publications on those versions of the MoCA to see if one of the authors was also the initial adaptor and could be contacted through the journal or via the corresponding author.

These questionnaires were then sent out to the adaptors via email, along with a request to check our English translation of their version. If the adaptors responded and agreed to complete the questionnaire, we sent a reminder email after 2 weeks. If the adaptors did not respond after another 2 weeks, the email request was re-sent. If no further reply was received after this, no further contact was made.

Overall, the contact information for six adaptors could not be identified (13% of questionnaires developed and 9.7% of all adaptations). Questionnaires for the remaining 39 versions were sent out. Of these, 17 were returned fully completed, including after follow-up emails (27.4% of all adaptations and 43.6% of questionnaires sent out).

### Step 3: collate data from steps 1 and 2 to form the guidelines

To develop guidelines for translating and culturally adapting the MoCA, we combined data on cultural adaptation and rationale behind the adaptation from our scoping review and questionnaire feedback from MoCA adaptors.

The questionnaires sent to adaptors were already organised by question and we were able to merge all adaptors’ feedback on the cultural adaptation procedures for each question. We again removed duplicating information so that each question of the MoCA had independent steps that could be undertaken to adapt it, along with the rationale.

For each question of the MoCA, its respective data on the cultural adaption steps and rationale behind cultural adaptation from the both the scoping review and questionnaire feedback were merged. Where we saw repetition (e.g. where the same concept or method for cultural adaption was being conveyed), the duplicate information was removed.

This resulted in a final list of mutually exclusive cultural adaptation steps for each question of the MoCA, along with rationale and guidance on how to conduct those steps. The respective papers and adapted versions of the MoCA were cited, resulting in a question-by-question set of guidelines.

## Results

### Step 1: scoping review

**Fig. 2:**
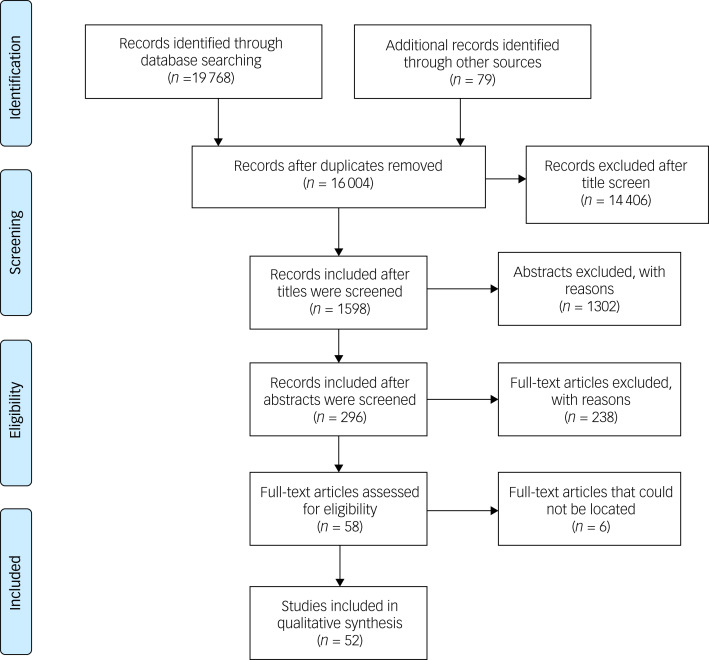
Flow diagram of the scoping review results.

Our search identified 19 768 papers on the MoCA (see Fig. 2 for the flow diagram). We then proceeded to screen the titles of the papers and excluded 14 406 papers. Following this, abstracts were screened, and this narrowed our search to 296 papers. The other 1302 papers were excluded as they only focused on the original English versions of the MoCA.

Full texts of the remaining 296 publications were screened and this brought the number of papers to 58. The remaining 238 papers were excluded as they were not the primary papers for their non-English version of the MoCA. For six of the papers, full-text articles could not be located despite contacting authors and the respective journals; details of these papers are available in the Supplementary Material. Therefore, we had 52 papers that were deemed suitable for analysis.

#### Reporting of the translation procedures and quality assessment of that reporting

The scoping review identified and considered seven discrete steps that can be undertaken in any order when translating the MoCA:

- Translation: The original English version is converted into the target language without any cultural adaptation. A native/fluent speaker of the language can be of help. Alternatively, an official translator may be used, e.g. ‘A Japanese geriatrician and a psychologist translated the original English version of the MoCA into Japanese’.^43^
- Back translation: An initial translation is converted back from the target language to English, e.g. ‘The revised Cantonese MoCA was then back-translated into English by another bilingual translator’.^44^
- Users in co-production: Individuals selected from the target population who may use the test give feedback or information that can influence the development of the test, e.g. ‘Twenty patients with confirmed stroke who were attending the outpatient physiotherapy clinic of the Universiti Kebangsaan Malaysia Medical Centre (UKMMC) participated. After completion of the BM [Bahasa Malaysia] MoCA, participants were asked to comment on whether any parts of the test were difficult to comprehend or complete.’^45^
- Expert recommendations: Individuals with specialist knowledge on translation, target languages or subject matters related to the test, provide information or advice that may affect how the translated test is developed, e.g. ‘The resulting revision was reviewed and amended by a translation committee, which consisted of 3 Korean psychiatrists and 2 psychologists.’^46^
- Revisions based on step-by-step feedback: Changes and adaptations are made to the translated test based on suggestions that have been proposed and approved, e.g. ‘Wording was adjusted where necessary, and the process was repeated until the final MoCA-ChLA [Chinese-Language Los Angeles] was considered by the two bilingual psychologists (ELT and PL) to be linguistically and semantically equivalent to the original MoCA, unambiguous, and easily comprehensible.’^47^
- Involvement of the original authors: Authors of the original test provide information or advice that may affect how the translated test is developed; in this instance, authors of the original paper by Nasreddine et al,^31^ e.g. ‘The original author (ZSN) reviewed and approved the back-translated MoCA-B [MoCA-Basic] version.’^48^
- Pilot study: A small scale preliminary study is implemented in which the translated version of the test is administered and evaluated to see whether it is viable and suitable for the proposed users, e.g. ‘A pilot study was conducted to establish whether the new BM [Bahasa Malaysia] version of the MoCA could be understood and completed by stroke patients in Malaysia.’^45^

Across the 52 papers in our scoping review, a total of 30 different languages were used, with Chinese being the most widely reported; eight papers were included that looked into the translation and cultural adaptation of the MoCA into a Chinese version. This was followed by five papers for the Turkish version, four papers for the Czech version, four papers for the Cantonese version, three papers for the Arabic version, two papers for the Finnish version, two papers for the Indonesian version, two papers for the Malay version and two papers for the Polish version. There was one paper each for the following MoCA versions; Brazilian, Chinese (Changsha), Dutch, English, Filipino, French, Georgian, German, Greek, Hebrew, Hiligaynon, Hungarian, Italian, Japanese, Kazakh, Korean, Malayalam, Persian, Portuguese, Sinhala, Slovenian and Swahili.

Out of 52 papers, 16 mentioned a translation process taking place but did not provide enough detail with regards to specific steps. Consequently, a score of 1 was given to these papers on the MTRQ scale. Of these, four papers were for the Turkish version,^49–52^ two were for the Chinese version^53,54^ and two were for the Czech version;^55,56^ there was one paper each for the Singaporean,^57^ Slovenian,^58^ Malay,^59^ Italian,^60^ Indonesian,^61^ Hungarian,^62^ Hebrew^63^ and Greek versions.^64^

Excluding the aforementioned papers, Table 2 shows the remaining 36 papers in which individual translation steps were reported. Of these, only four papers (Chu et al,^44^ Freitas et al,^90^ Husein et al^84^ and Sahathevan et al^45^) reported using all of the translation steps. The remaining 32 reported to have undertaken some translation steps, in various combinations.

**Table 2:** All papers included in our analysis, with the reported translation steps undertaken

Papers	Year	Language	Country	MTRQ score	MCAR score	Translation	Back translation	Users in co-production	Expert recommendations	Revisions based on step-by-step feedback	Involvement of original authors	Pilot study
Benabdeljlil et al^65^	2017	Arabic	Morocco	2a	2a	*						*
Hayek et al^66^	2020	Arabic	Lebanon	2a	4a	*			*			
Rahman and El Gaafary^67^	2009	Arabic	Egypt	2a	2a	*	*					*
Memoria et al^68^	2012	Brazilian	Brazil	4a	0	*	*	*	*			
Chu et al^44^	2015	Cantonese	Hong Kong	4a	4a	*	*	*	*	*	*	*
Wong et al^33^	2009	Cantonese	Hong Kong	4a	4a	*	*		*	*	*	
Wong et al^69^	2018	Cantonese	Hong Kong	2a	2a	*			*		*	
Chang et al^53^	2012	Chinese	Taiwan	1	2a	*						
Chen et al^48^	2016	Chinese	China	2a	1	*	*				*	
Hu et al^54^	2013	Chinese	China	1	4a	*						
Nie et al^70^	2012	Chinese	China	2a	2a	*			*		*	
Tsai et al^71^	2012	Chinese	Taiwan	4a	2a	*	*		*	*	*	
Yu et al^72^	2012	Chinese	China	2a	2a	*						
Zheng et al^47^	2012	Chinese	USA	4a	4a	*	*	*	*	*		
Li et al^73^	2020	Chinese (Changsha)	China	2a	1	*			*			
Dong et al^57^	2010	Chinese	Singapore	1	4a	*						
		English										
		Malay										
Bartos et al^74^	2014	Czech	Czech Republic	4a	2a	*	*			*	*	
Bezdicek et al^55^	2010	Czech	Czech Republic	1	0	*						
Bezdíček et al^75^	2019	Czech	Czech	3	4a	*					*	
Reban^56^	2006	Czech	Czech Republic	1	0	*					*	
Thissen et al^76^	2010	Dutch	Holland	4a	0	*	*					
Dominguez et al^77^	2013	Filipino	Philippines	4a	1	*	*		*	*		*
Nortunen et al^78^	2018	Finnish	Finland	2a	0	*	*					
Puustinen et al^79^	2016	Finnish	Finland	2a	0	*	*					
Nasreddine and Patel^80^	2016	French	Canada	2a	2a	*				*	*	*
Janelidze et al^81^	2017	Georgian	Georgia	4b	4b	*						
Costa et al^82^	2012	German	Germany	4a	4a	*	*		*			
Konstantopoulos et al^64^	2016	Greek	Greece	1	1	*						
Lifshitz et al^63^	2012	Hebrew	Israel	1	1	*						
Aliling et al^83^	2019	Hiligaynon	Philippines	4b	3	*	*	*	*	*		*
Volosin et al^62^	2013	Hungarian	Hungary	1	0	*						
Husein et al^84^	2010	Indonesian	Indonesia	4b	2b	*	*	*	*	*	*	*
Panentu and Irfan^61^	2013	Indonesian	Indonesia	1	1	*						
Pirrotta et al^60^	2015	Italian	Italy	1	1	*						
Fujiwara et al^43^	2010	Japanese	Japan	4a	4a	*	*		*	*		*
Tsoy et al^85^	2019	Kazakh	Kazakhstan	2a	0	*						
Lee et al^46^	2008	Korean	South Korea	4a	4a	*	*		*	*		*
Razali et al^59^	2014	Malay	Malaysia	1	1	*						
Sahathevan et al^45^	2014	Malay	Malaysia	4a	2a	*	*	*	*	*	*	*
Krishnan et al^86^	2015	Malayalam	India	4a	2a	*	*		*		*	
Emsaki et al^87^	2011	Persian	Iran	2a	2a	*						
Gierus et al^88^	2015	Polish	Poland	4a	4a	*	*				*	
Magierska et al^89^	2012	Polish	Poland	4a	4a	*	*					
Freitas et al^90^	2010	Portuguese	Portugal	4a	4a	*	*	*	*	*	*	*
Karunaratne et al^91^	2011	Sinhala	Sri Lanka	4a	4a	*	*		*			*
Potocnik et al^58^	2020	Slovenian	Slovenia	1	0	*						
Vissoci et al^92^	2019	Swahili	Tanzania	4b	1	*	*	*	*	*		*
Krist et al^50^	2019	Turkish	Germany	1	2a	*						
Aksoy et al^49^	2013	Turkish	Turkey	1	0	*						
Ozdilek and Kenangil^51^	2014	Turkish	Turkey	1	2a	*						
Selekler et al^93^	2010	Turkish	Turkey	4a	4a	*	*		*			*
Yancar and Özcan^52^	2015	Turkish	Turkey	1	1	*						

MTRQ, Manchester Translation Reporting Questionnaire; MCAR, Manchester Cultural Adaptation Reporting Questionnaire.

All of the papers reported simple translation, 25 papers undertook back translation, 21 involved the use of expert recommendations, 15 involved original authors, 14 made revisions based on step-by-step feedback, 14 directed pilot studies and eight reported users in co-production.

#### Reporting of the cultural adaptation procedures and quality assessment

Out of the 52 papers, ten (Aksoy et al,^49^ Bezdicek et al,^55^ Memoria et al,^68^ Nortunen et al,^78^ Potocnik et al,^58^ Puustinen et al,^79^ Reban,^56^ Thissen et al,^76^ Tsoy et al^85^ and Volosin et al^62^) did not mention any cultural adaptation that may have taken place, resulting in a score of 0 on the MCAR scale. Ten papers (Chen et al,^48^ Dominguez et al,^77^ Konstantopoulos et al,^64^ Li et al,^73^ Lifshitz et al,^63^ Panentu et al,^61^ Pirrotta et al,^60^ Razali et al,^59^ Vissoci et al^92^ and Yancar and Özcan^52^) mentioned that a cultural adaptation process took place but did not describe it, scoring 1 on the MCAR.

For the remaining 32 papers, Table 3 highlights which questions were reportedly culturally adapted. Furthermore, this shows the regularity with which each question needed to be adapted across the papers. Question 10 for language was most frequently culturally adapted, in 20 out of 32 papers. This was closely followed by question 5 (in 19 out of 32 papers), and by extension, question 12. Question 9 for language was found to be culturally adapted in 15 papers.

**Table 3:** The frequency of cultural adaptation across questions of the Montreal Cognitive Assessment in papers

		Montreal Cognitive Assessment questions												
Authors	Language	1. Executive functioning	2. Visuospatial abilities	3. Visuospatial abilities	4. Language – naming	5. Memory – anterograde	6. Attention – digit span	7. Attention – vigilance	8. Attention – serial 7s	9. Language – repetition	10. Language – fluency	11. Abstraction	12. Memory – recall	13. Attention – orientation
Benabdeljlil et al^65^	Arabic	*				*		*		*	*		*	
Hayek et al^66^	Arabic					*					*		*	
Rahman and El Gaafary^67^	Arabic									*				
Chu et al^44^	Cantonese	*				*					*	*	*	*
Wong et al^33^	Cantonese	*						*			*			*
Wong et al^69^	Cantonese	*	*	*	*	*	*	*	*	*	*	*	*	
Chang et al^53^	Chinese	*						*			*			
Hu et al^54^	Chinese	*						*		*	*			
Nie et al^70^	Chinese	*						*			*			
Tsai et al^71^	Chinese	*						*			*			*
Yu et al^72^	Chinese	*						*			*			
Zheng et al^47^	Chinese					*					*		*	*
Dong et al^57^	Chinese	*			*	*		*		*	*		*	
	English													
	Malay													
Bartos et al^74^	Czech									*				
Bezdíček et al^75^	Czech										*			
Nasreddine and Patel^80^	French				*	*				*	*	*	*	
Janelidze et al^81^	Georgian									*	*			
Costa et al^82^	German										*			
Krist et al^50^	German					*					*		*	
	Turkish													
Aliling et al^83^	Hiligaynon				*	*					*		*	
Husein et al^84^	Indonesian				*	*				*			*	
Fujiwara et al^43^	Japanese					*							*	
Lee et al^46^	Korean	*				*					*		*	
Sahathevan et al^45^	Malay									*				
Krishnan et al^86^	Malayalam					*				*			*	
Emsaki et al^87^	Persian					*				*			*	
Gierus et al^88^	Polish		*			*							*	
Magierska et al^89^	Polish									*				
Freitas et al^90^	Portuguese					*				*			*	
Karunaratne et al^91^	Sinhala	*			*	*		*		*	*		*	
Ozdilek and Kenangil^51^	Turkish					*							*	
Selekler et al^93^	Turkish					*							*	

Question 1 for executive functioning was culturally adapted in 12 papers, question 7 for attention was adapted in ten papers, question 4 for language was adapted in six papers, question 13 for attention was adapted in five papers and question 11 for abstraction was adapted in three papers. Only two papers culturally adapted question 2 for visuospatial abilities, and only one paper each culturally adapted question 3 for visuospatial abilities, question 6 for attention and question 8 for attention.

This highlights which cognitive domains, and their respective questions, are dependent on cultural knowledge, and which rely on a simple translation.

#### Individuals involved in translation and cultural adaptation

Of the 52 papers that mentioned translation and cultural adaptation processes in some level of detail, 21 publications elaborated further the individuals involved in the translation and cultural adaptation process (see Table 4).

**Table 4:** Reported individuals involved in translation and cultural adaptation

Papers	Language	Bilingual/native- speaking translator	Geriatrician	Psychiatrist	Psychologist	Occupational/ speech and language therapist	Neurologist/ physician	Nurse	Linguistics expert	Bilingual experts/ researchers	Investigators
Hayek et al^66^	Arabic								*	*	
Memoria et al^68^	Brazilian	*			*		*				
Chu et al^44^	Cantonese	*	*	*	*	*					
Wong et al^33^	Cantonese				*		*				
Wong et al^69^	Cantonese				*	*	*				
Nie et al^70^	Chinese			*							
Tsai et al^71^	Chinese			*			*				
Zheng et al^47^	Chinese				*					*	
Li et al^73^	Chinese (Changsha)									*	
Bartos et al^74^	Czech	*									
Dominguez et al^77^	Filipino		*	*	*	*	*	*			
Costa et al^82^	German	*			*		*				
Husein et al^84^	Indonesian	*					*		*		*
Fujiwara et al^43^	Japanese		*	*	*		*			*	
Lee et al^46^	Korean			*	*		*				
Sahathevan et al^45^	Malay	*								*	
Krishnan et al^86^	Malayalam				*					*	*
Freitas et al^90^	Portuguese	*			*				*		
Karunaratne et al^91^	Sinhala									*	
Vissoci et al^92^	Swahili	*						*		*	*
Selekler et al^93^	Turkish									*	

Of these, 11 mentioned psychologists, nine mentioned neurologists/physicians, eight mentioned bilingual experts/researchers, eight referred to bilingual/native-speaking translators and six referred to psychiatrists. Three papers mentioned geriatricians, three mentioned occupational/speech and language therapists, three referred to a linguistics expert and three mentioned the use of an investigator. Only two papers mentioned nurses.

### Step 2: feedback from previous MoCA adaptors

Table 5 summarises which questions of the MoCA were culturally adapted by adaptors, thus showing the frequency of cultural adaptation undertaken for each question. The table also highlights which adaptors returned feedback that provided rationale for their cultural adaptation.

**Table 5:** Questionnaires distributed to adaptors of the Montreal Cognitive Assessment

Language	Montreal Cognitive Assessment domain													Papers referenced
	1. Executive functioning	2. Visuospatial abilities	3. Visuospatial abilities	4. Language – naming	5. Memory – anterograde	6. Attention – digit span	7. Attention – vigilance	8. Attention – serial 7s	9. Language – repetition	10. Language – fluency	11. Abstraction	12. Memory – recall	13. Attention – orientation	
Afrikaans					*				*	*		*		
Bulgarian							*		*	*			*	
Chinese (Changsha)				*										
Chinese (Hong Kong)^a^							*		*	*				Yes
Chinese (Mandarin)^a^					*		*		*	*		*		No
Chinese (Singapore)	*			*	*		*		*	*		*	*	
Chinese (Taiwan)^a^					*		*		*	*		*		Yes
Creole^a^					*				*			*		Yes
Croatian^a^										*				Yes
Czech									*	*				
Danish					*							*	*	
Dutch^a^									*	*				Yes
English (Singapore)	*			*	*		*		*	*		*	*	
Estonian					*				*	*		*		
Filipino^a^				*	*				*	*		*		Yes
Finnish^a^					*				*	*		*		Yes
French									*					
Georgian					*				*	*		*		
German					*				*			*		
Greek^a^										*				No
Hungarian					*				*	*		*		
Italian^a^									*					Yes
Japanese^a^					*				*	*		*		Yes
Korean^a^					*				*	*		*		Yes
Latvian					*				*	*		*		
Malay (Bahasa Malaysia)^a^										*				Yes
Malay (Singapore)	*			*	*		*		*	*		*	*	
Norwegian^a^									*					No
Persian					*				*	*		*		
Polish									*					
Portuguese^a^					*				*	*		*		Yes
Punjabi				*										
Romanian									*					
Serbian^a^					*					*	*	*		No
Spanish					*				*	*		*		
Swedish					*				*			*		
Turkish					*				*	*		*		
Urdu^a^					*				*	*		*		Yes
Welsh					*					*		*		

* Question was culturally adapted.

a. Questionnaire was returned.

We can see that the majority of adaptors culturally adapted question 5, and by extension question 12, for memory, and questions 9 and 10 for language. We can also see that some adaptors culturally adapted question 4 for language, question 7 for attention and question 13 for orientation. Only three adaptors culturally adapted question 1 for executive functioning, and only one adapted question 11 for abstraction.

In contrast, none of the adaptors had culturally adapted questions 2 and 3 for visuospatial abilities, and questions 6 and 8 for attention that pertains to numbers.

Table 6 provides examples of some of the modifications adaptors reported back in the questionnaires along with the rationale they provided for some of these questions.

**Table 6:** Cultural adaptation of Montreal Cognitive Assessment items from feedback provided through questionnaires

Assessment question	Example of adapted version	Language of version	Rationale
4. Language – naming: Show image of rhinoceros and ask them to name it.	Image of a ‘rhinoceros’ was replaced with an image of an ‘owl’	Filipino	Based on pilot studies, elderly patients were unable to name a rhinoceros as it is not typically in their vocabulary. The owl, which is an indigenous Philippine fauna used with medium frequency and is distinctly identifiable by its prominently big round eyes, was chosen instead
5. Memory – anterograde: Repeat and remember the word velvet, daisy and red	Velvet was changed to linen, daisy was changed to clove and red was changed to blue. The following word was substituted: ‘face’	Portuguese	The words were chosen based on a series of familiarity in the culture, retaining semantic equivalence, similar number of syllables and similar word frequency in the language
7. Attention – vigilance: Read a list of letters and tap your hand each time the letter A appears.	The letters were changed to numbers	Chinese (Hong Kong, Mandarin, Taiwan)	Chinese is a character-based language that does not use the alphabet
9. Language – repetition: Repeat the sentences ‘I only know that John is the one to help today’	The name ‘John’ changed was changed to ‘Abdullah’	Urdu	‘John’ is a very uncommon name in this language. The name chosen is a prevalent cultural equivalent to the name ‘John’ and is well-known among elderly populations
10. Language – fluency: List as many words as you can starting with the letter F in 1 minute.	Changed from a letter fluency to a category fluency task, i.e. name as many fruits, vegetables or animals as you can	Korean	Korean is a character-based language. The task was adjusted to a category fluency task as it can achieve the same purpose as the original task, which is to reflect executive function

As with our scoping review, this has highlighted cognitive domains, and their respective questions, dependent on cultural knowledge.

We also found that of the 17 returned questionnaires, 13 of the adaptors mentioned their adapted MoCAs being mentioned in publication; these were screened and added to the scoping review retroactively.

### Step 3: collate data from steps 1 and 2 to form the guidelines

**Fig. 3:**
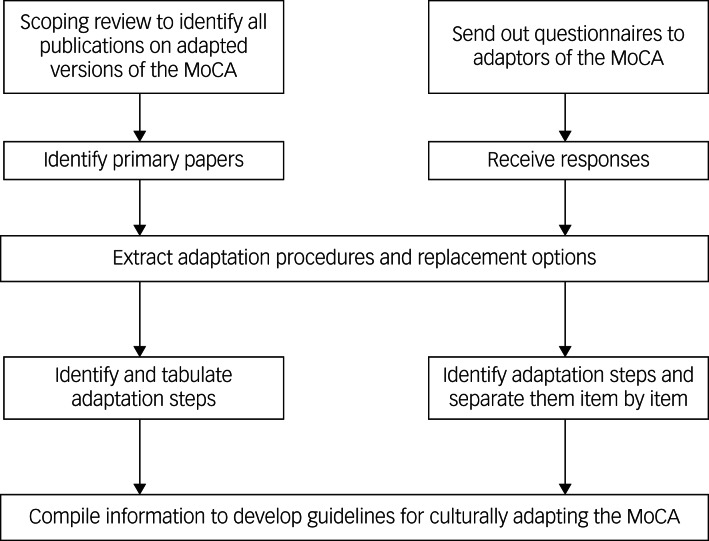
Development process of the guidelines.

For each question of the MoCA, the individual cultural adaptation steps identified from our scoping review and from adaptors’ feedback, along with the rationale for undertaking cultural adaptation, were tabulated to form the guidelines. Figure 3 summarises the steps and processes we undertook to synthesise the data into guidelines for the adaptation of the MoCA.

For each question, the following was presented:

- How the questions have been previously culturally adapted, with steps undertaken.
- Citing of the respective languages and adaptors that used these steps.
- The rationale behind adapting the question with these steps and the replacement chosen.

**Fig. 4:**
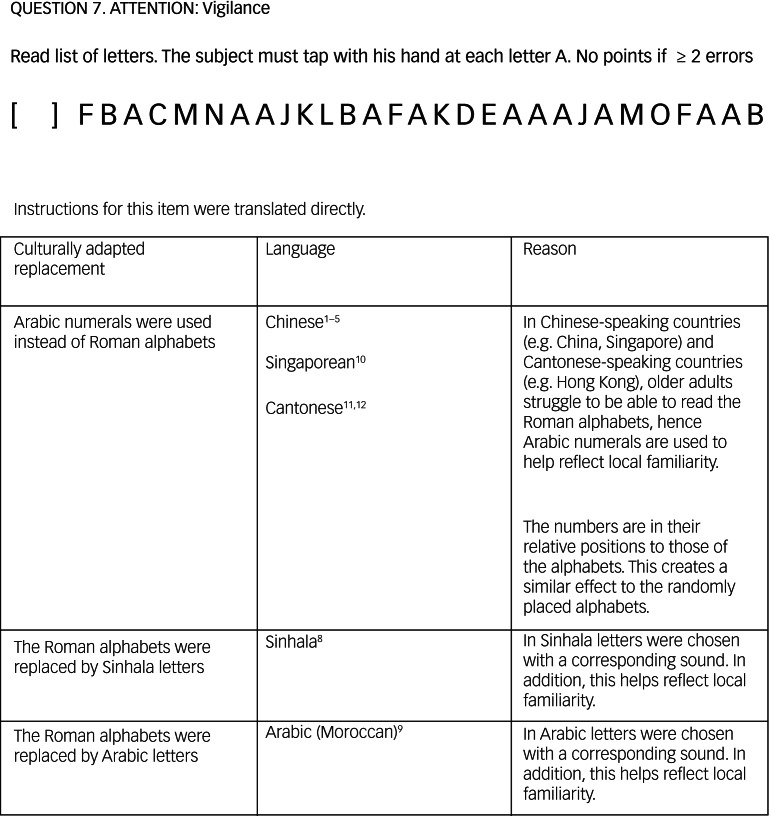
Example from the guidelines.

Figure 4 shows an excerpt from the guidelines, providing examples of these.

The full guidelines are 29 pages providing data and guidance on the translation and cultural adaptation of the MoCA. These guidelines display information spanning 32 languages and 38 countries (see Supplementary Appendix 2 for a copy of the guidelines).

## Discussion

The diagnosis of dementia and MCI within elderly ethnic minority populations is currently compromised because of the lack of cognitive tests that account for the language and culture of individuals from these populations.^19,20^ The current breadth of cognitive tests are susceptible to cultural bias and require an extensive cultural adaptation beyond a translation verbatim.^25,26,30^

One solution to this is to produce individual guidelines for the cultural adaptation of various cognitive tests, as has been previously done for the ACE-III.^36,38^ We aimed to replicate these methods for the MoCA, a popular cognitive test used to screen and diagnose for dementia and MCI. Through the combination of a scoping review and questionnaire feedback from adaptors of the MoCA, we were able to successfully develop guidelines on translating and culturally adapting the MoCA.

The scoping review was able to list all papers on existing translations and cultural adaptation of the MoCA and through the MTRQ and MCAR ratings, highlight which papers provide the most replicable data on translating and culturally adapting the MoCA for future adaptors to consider.

Through our review, a full list of independent translation steps that can be undertaken by future adaptors was identified along with the frequency of their occurrence. This allows future adaptors to make judgement calls regarding which steps they may endeavour to undertake, based on what previous adaptors followed. Naturally, some approaches such as simply having a native speaker back-translate a measure would be problematic from a clinical or psychometric standpoint.^94^ A native speaker is not necessarily someone qualified or informed about the nuances of cognitive tests, thus an additional expert in this field of study would be desirable to help form a cultural adaptation or accurate translation.

We can see that direct translation was the most common step, undertaken by all of the publications, followed by back translation, expert recommendations, the involvement of original authors, revisions based on step-by-step feedback, pilot studies and finally, users in co-production. Across the same language versions, although there is some overlap of translation steps undertaken, there is no consistency. For example, in one Indonesian version all of the translation steps were undertaken,^84^ whereas in the other only one step was.^61^ This would suggest that translation steps were undertaken on the basis of convenience and resources, as opposed to the best fit for different language and cultural groups.

We also identified which cognitive domains and their respective questions were considered to be dependent on culture and therefore most likely to be culturally adapted. Question 10 for language, which focused on verbal fluency, was culturally adapted the most, followed by further questions for language and memory. On the other hand, questions for attention based on numbers (i.e. serial 7s and digit span) and visuospatial abilities had not been reportedly culturally adapted as much in any of the papers.

Thus, we have identified which possible domains of the MoCA display significant cultural reliance. It is important to note that although cognitive domains such as attention and visuospatial abilities were not frequently adapted in our findings, this does not necessarily mean that they cannot be influenced by culture in other cases.

Through our questionnaires we were able to acquire additional and far richer data on the cultural adaptation of the MoCA, including rationale behind changes made. As with our scoping review, this feedback from adaptors showed which questions were most likely to require cultural adaptation, indicating their cultural dependence.

Findings from the questionnaires mirrored those from our review: question 10 for language was once again the most culturally adapted, followed by the same questions for language and memory. Similarly, questions for attention based on numbers, executive functioning and visuospatial abilities received little to no cultural adaptation by adaptors.

The previous systematic review of adaptations of the ACE-III reported similar findings across cognitive domains and their questions. Reasoning behind these findings in both our scoping review and across questionnaires is that executive functioning, visuospatial abilities and number-based tasks are not associated with culture. This indicates to future adaptors which questions should be prioritised for cultural adaptation.

Across the same language versions there was little overlap, suggesting that different cultures may have different needs with regards to cultural adaptation, even if they share a language. Interestingly, however, within the Cantonese and Chinese versions there was consistency across the versions in requiring cultural adaptation for question 1 for executive functioning and question 7 for attention – questions that typically do not require adaptation. This is because they rely specifically on the alphabet, in contrast to Cantonese and Chinese characters. In this instance, language and culture played a dual role and required the cultural adaptation of these questions for these specific language versions, irrespective of which country the version was for.

Our scoping review was restricted in several ways that prevented the incorporation of some language and cultures. First, it was restricted by what was reported in the papers. Further and additional adaptation procedures that may have been undertaken by adaptors but were not mentioned in their respective papers still resulted in scores of 1 and 0 on the MTRQ and MCAR. The scales also cannot guarantee the quality of those processes, and whether they resulted in robustly adapted versions of the MoCA.

Second, regarding our questionnaires sent to adaptors, we were limited by resources and translators available to us, and therefore unable to produce and distribute questionnaires for all 62 versions of the MoCA. The relatively poor return rate of 17 out of 62 questionnaires reduced the amount of additional information we could have included in the guidelines.

This highlights the significance of conducting the scoping review and questionnaires in tandem, and combining the findings to allow for the incorporation of as much information as possible. The guidelines are not restricted to information available in published literature, and incorporate primary experiences of cultural adaptation by existing adaptors of the MoCA, which accounted for adaptations that may not have had corresponding papers.

Additionally, the adaptations reported were culturally skewed favouring primarily MoCA versions from Asia (mainly China) and Europe. However, the guidelines are designed in such a way that they can be used to culturally adapt the MoCA in countries such as those from Africa or South America.

If a language in these countries is not letter-based but character-based, then it may be more appropriate to use a category fluency task rather than a letter fluency task, as it has been shown to be just as effective. Similarly, if the images of animals that have to be recognised are not animals familiar in certain countries, native animals knowledgeable to older groups should be selected. Words that have to be remembered should not just be directly translated into the corresponding language; a word that represents the same concept but also has the same fluency in the target language and, ideally, the same number of syllables should be used instead.

Examples and associated rationale like this within the guidelines can help support and guide adaptors who want to adapt the MoCA for their respective language and culture, regardless of where they are from.

Overall, we replicated a novel approach that has only previously been done once before. However, unlike the ACE-III guidelines, for the MoCA we were able to gain more data as the MoCA originated earlier, with far more translated and culturally adapted versions. Although the ACE-III guidelines compiled data from 22 languages and cultural contexts,^38^ the guidelines for the MoCA compiled data for 32 languages across 38 countries. Additionally, the ACE-III review found 32 papers and only six completed questionnaires from adaptors of the ACE-III were received.^36^ In comparison, the MoCA guidelines are composed of far richer and extensive data.

The implications of this methodology, and the findings regarding questions pertaining to specific cognitive domains, can be replicated further to develop guidelines in the same manner for other cognitive tests. Furthermore, these particular methods allow for familiarity with a chosen test.

However, although we have demonstrated that these set of guidelines can assist future adaptors in creating their own version of the MoCA, it does not obviate the need to conduct a formal cultural and psychometric validation within a target population. These are essential steps that should follow the development and utilisation of guidelines to produce a culturally adapted version of a test.

Naturally, other factors beyond culture, such as age and education level, can also influence sensitivity. Therefore, it may also be appropriate to use cut-offs and point adjustments for these factors once translation and cultural adaptation has occurred for a test, and future researchers should endeavour to establish what these might be during psychometric validation in large clinical populations.

Overall, these guidelines can act as a starting point for future adaptors of the MoCA, and have been designed so that necessary updates and further information on cultural adaptation can continue to be added to them. The next step would be to implement these guidelines to develop an adaptation of the MoCA for a particular language and cultural context, as was done for the ACE-III, to demonstrate usability and cultural validity.

## Data Availability

The data that support the findings of this study are available from the corresponding author, N.M., upon reasonable request.

## Supplementary material

For supplementary material accompanying this paper visit http://doi.org/10.1192/bjo.2021.1067. click here to view supplementary material
