# Supplementary material for: Developing guidelines for the translation and cultural adaptation of the Montreal Cognitive Assessment: scoping review and qualitative synthesis
Source: BJPsych Open. 2022 Jan 7;8(1):e21. doi: 10.1192/bjo.2021.1067 (PMC8811786; doi:10.1192/bjo.2021.1067)
Supplement: Supplementary file 1 [file bjosup.zip › S205647242101067Xsup001.pdf]

MANCHESTER 1824
The University of Manchester

DOME Project
Dementia in Ethnic Minorities

GUIDELINES ON TRANSLATING AND CULTURALLY ADAPTING

THE MONTREAL COGNITIVE ASSESSMENT (MOCA)

Developed at the Centre for Primary Care and Health Services Research(2020)

GHAZN KHAN
NADINE MIRZA
WAQUAS WAHEED

CONTENTS

Acknowledgements & Copyright	2
Translation Steps	3
Cultural Adaptation	4
Question1: Executive functioning	5
Question 2: Visuospatial Abilities	6
Question 3: Visuospatial Abilities	7
Question 4: Language- Naming	8
Question 5: Memory- Anterograde	10
Question 6: Attention- Digit Span	15
Question 7: Attention- Vigilance	16
Question 8: Attention- Serial 7s	17
Question 9: Language- Repetition	18
Question 10: Language- Fluency	21
Question 11: Abstraction	24
Question 12: Memory- Recall	25
Question 13: Attention- Orientation	26
References	27

ACKNOWLEDGEMENTS

Many thanks to *Kathleen Gallant* and *Ziad Nasreddine* from mocatest.org in providing access to the MoCA rights to conduct research with the MoCA and information and support for contact adaptors of the MoCA.

Many thanks also to *Adrian Wong, Agogiatou Christina and Magda Tsolaki, Alessandro Pirani, Elaine Chan, Farrukh Habib, Hiroyuki Suzuki and Momoko Kobayashi and Yoshinori Fujiwara, Irena Martinic Popovic, Jacqueline C. Dominguez, Jeannette Moreira and Luis Faisca, Jos F M de Jonghe, Jukka Puustinen, Jun-Young Lee, Marleen Regina van Walsem, Pengxu Wei, Ramesh Sahathevan, Sandra Freitas,* and *Vanja Kljajevic* for their responses to questionnaires that enquired about the cultural adaptation process they undertook which informed these guidelines.

COPYRIGHT

Dr. Ziad Nasreddine is the copyright holder of the MoCA. Any proposed changes or translations developed, including those aided by these guidelines, must be submitted for approval to *Dr. Nasreddine*, at CEDRA Center for Diagnosis and Research on Alzheimer's Disease, prior to implementation (info@mocatest.org).

For further information regarding these guidelines please contact *Nadine Mirza* (nadine.mirza@postgrad.manchester.ac.uk).

TRANSLATION STEPS

The items of the MoCA will require translation into a target language. The following summarises the translation steps that have been undertaken by previous adaptors of the MoCA.

1. Translation: Direct translation, without any form of cultural adaptation, from English into the target language, often with the assistance of a native or fluent speaker of the language or an official translator.

2. Back Translation: Creating a retroversion of the initial translation, from the target language back to English, often with the assistance of a native or fluent speaker of the language or an official translator.

3. Users in Coproduction: Potential or future users of the assessment, including native and fluent speakers of the language, providing feedback or information in any way that influences the development of the translated assessment.

4. Expert Recommendations: Experts on translation, the target languages, or subject matters related to the assessment providing feedback or information in any way that influences the development of the translated assessment.

5. Revisions based on step-by-step feedback: Constant and continuous revisions of the translated assessment informed by feedback as soon as it is presented.

6. Involvement of the original authors: Authors of the original assessment providing feedback or information in any way that influences the development of the translated assessment.

7. Pilot Study: Administering translated versions of the assessment.

In addition, a skilled team can be recruited from **Mapi Language services**, who can validate the linguistic adaptation of the MoCA into a particular language. They conduct the following processes on the linguistically adapted MoCA;

i. Update - by a native speaker to bring existing version in line with an upgraded original version
ii. Backtranslation step – 1 backtranslation by a qualified translator
iii. Clinician's Review step
iv. Proofreading step

The above information can be found on www.mocatest.org

CULTURAL ADAPTATION

The items of the Montreal Cognitive Assessment (MoCA) that require cultural adaptation are identified in these guidelines. These guidelines detail how each item has been previously culturally adapted across several languages in the MoCA. Following the reasoning and procedures detailed below, an item-by-item cultural adaptation of the MoCA can be conducted.

It is also worth noting that these guidelines are primarily based on an adaptation of the MoCA version 7.1. However, we have mentioned how alternate versions have been adapted (marked in **bold** and *).

QUESTION 1. VISUOSPATIAL/EXECUTIVE FUNCTIONING: Alternate trail making

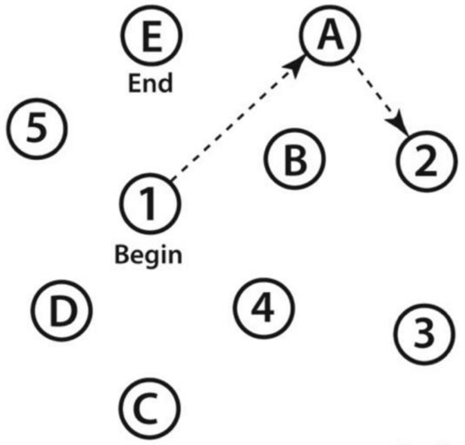

Often, the item is directly translated however some cultural adaptation may be necessary.

Culturally adapted replacement	Language	Reason
English alphabet letters are substituted with letters/characters of chosen language	Chinese[1–5], Cantonese[6], Korean[7], Sinhala[8], Arabic (Moroccan)[9]	Older adults in some countries struggle to read the English alphabet and this must be substituted with characters of the local language.<br><br>Common serial words in Chinese 甲, 乙, 丙, 丁 and 戊 can be used instead, as they are the equivalent to A,B,C,D and E<br><br>The English alphabet was replaced with Cantonese words<br><br>The English alphabet was replaced with Korean words.<br><br>The English alphabet was replaced with Sinhala words.
Alternating shapes from circle to triangle replaced Roman alphabets	English (Singapore)/Chinese/Malay[10]	There are no substitutes for Roman alphabets in some variations of Chinese.
The Colour Trails Test 2 (CTT-2)/ shape trails test replaced the Alternate Trail Making task.	Cantonese[11,12]	There are no substitutes for Roman alphabets in the Cantonese variation of Chinese.

QUESTION 2. VISUOSPATIAL/EXECUTIVE FUNCTIONING: Wire Cube

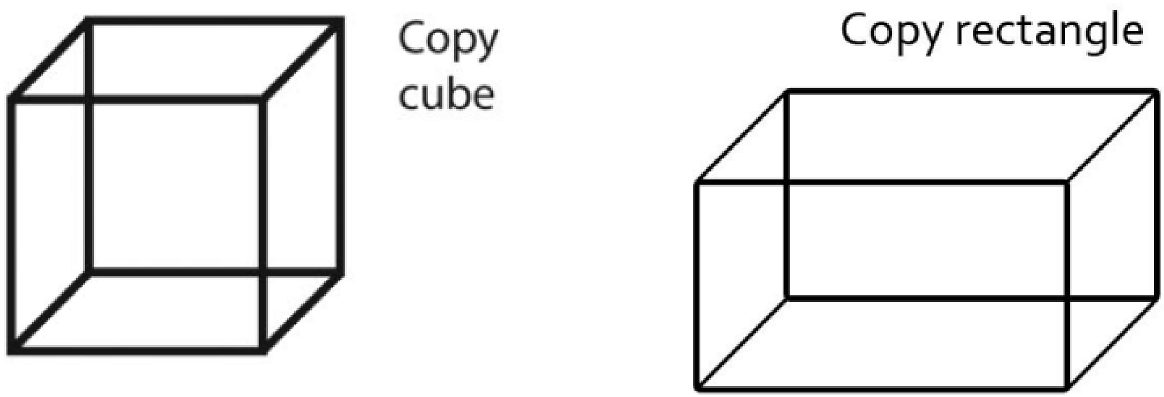

Figure 1. Version 7.2

Often, the item is directly translated however some cultural adaptation may be necessary (this is particularly for the Polish version 7.2).

Culturally adapted replacement	Language	Reason
Cube replaced by cuboid<br><br>Place the command “Copy shape” in the sheet instead of ‘‘Copy rectangle’’,	Cantonese[12]<br><br>Polish*[13]	The cuboid was chosen as a replacement as it retained domain specificity and is relevant to the cultural and demographic background of the intended users.<br><br>The original version of the task in the Polish suggests a plane figure – rectangle- which can be confusing to the subject and cause them to fail the task.<br><br>The task “Copy cuboid” could be too complicated, especially for respondents with fewer years of formal education.

3. VISUOSPATIAL/EXECUTIVE FUNCTIONING: Clock

Draw CLOCK (Ten past eleven)

Culturally adapted replacement	Language	Reason
The time was set to:<br><br>“Ten past six”/ “Ten past four”	Cantonese[12]	In Cantonese-Hong Kong versions the times “Ten past six”/ “Ten past four” were chosen as they retain domain specificity and are relevant to the cultural and demographic background of the intended users.

QUESTION 4. LANGUAGE: Naming

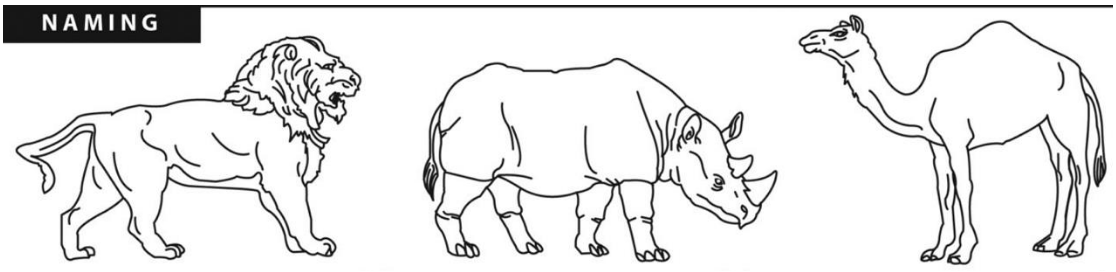

Often, the item is directly translated however some cultural adaptation may be necessary.

Culturally adapted replacement	Language	Reason
The ''Lion'' was replaced with a:<br><br>**Snake** in version 7.2<br><br>**Horse** in version 7.3<br><br>Panda<br><br>Elephant	**French***[14]<br><br>Cantonese[12]<br><br>Indonesian[15]	In the French versions 7.2 and 7.3; lion, rhino, and camel were replaced with similarly complex and frequently known animals (highlighted in **bold).**<br><br>In Cantonese-Hong Kong versions the ''Panda'' is a similarly complex and frequently known animal comparable to the ''Lion'', whilst also maintains domain specificity.<br><br>Cognitively healthy people in Indonesia had difficulty in identifying a "Lion"; "Elephant" was chosen as it reflects local familiarity.
The ''Rhinoceros" was replaced with a:<br><br>Rabbit<br><br>Owl<br><br>**Elephant** in version 7.2<br><br>**Tiger** in version 7.3<br><br>Elephant/ Deer	Sinhala[8]<br><br>English (Singapore)/Chinese/Malay[10]<br><br>Filipino, Hiligaynon[16]<br><br>**French***[14]<br><br>Cantonese[12]	Cognitively healthy people in Sri Lanka had difficulty in identifying a "Rhinoceros".<br><br>In Singapore the "Rhinoceros" was replaced by the "Elephant" to help reflect local familiarity<br><br>Elderly in the Filipino speaking population had difficulty recognising a "Rhinoceros". ''Owl'' was the replacement as it is indigenous to the Philippines and is used with medium frequency. A larger proportion of Filipino speaking adults were able to

		recognise it. Similarly, in Indonesia, "Owl" is far more familiar than "Rhinoceros".<br><br>In the French versions 7.2 and 7.3; "Lion" with a similarly complex and frequently known animals (highlighted in **bold)**<br><br>In Cantonese-Hong Kong versions the ''Elephant'' or ''Deer'' are similarly complex and frequently known animals comparable to the ''Rhinoceros'', whilst also maintaining domain specificity
The ''Camel'' was replaced with a:<br><br>Elephant<br><br>**Crocodile** in version 7.2<br><br>**Duck** in version 7.3<br><br>Zebra/ Squirrel	Sinhala[8]<br><br>**French***[14]<br><br>Cantonese[12]	Cognitively healthy people in Sri Lanka had difficulty in identifying a "Camel".<br><br>In the French versions 7.2 and 7.3; "Camel" was replaced with similarly complex and frequently known animals (highlighted in **bold)**.<br><br>In Cantonese-Hong Kong versions the ''Zebra'' or ''Squirrel'' are similarly complex and frequently known animals comparable to the ''Camel'', whilst also maintaining domain specificity.

QUESTION 5. MEMORY: Recall

MEMORY Read list of words, subject must repeat them. Do 2 trials, even if 1st trial is successful. Do a recall after 5 minutes.

	FACE	VELVET	CHURCH	DAISY	RED
1st trial					
2nd trial					

	TRUCK	BANANA	VIOLIN	DESK	GREEN
1st trial					
2nd trial					

Figure 2. Version 7.2

Instructions for this item were translated directly.

Culturally adapted replacement	Language	Reason
The word ''Face'' was replaced with:<br><br>**Hand** in version 7.2 and **Leg** in version 7.3<br><br>Nose<br><br>Hair<br><br>Arm	**French***[14]<br><br>Turkish[17–19], Finnish<br><br>Chinese-Language Los Angeles[20], Cantonese[12]<br><br>Cantonese[12]	The category, frequency, and length should be retained.<br><br>For the French versions 7.2 and 7.3 the category, frequency, and length of the words in version 7.1 were respected.<br><br>In Turkish "Face" has many meanings, hence confusion can be caused. Therefore, ''Face'' was replaced with ''Nose''.<br><br>In the Cantonese "Face" could mean the physical face or the social face. ''Hair'' is an unambiguous 2-character word in Chinese/Cantonese and familiar to all.<br><br>''Arm'' is also an unambiguous 2-character word in Cantonese and familiar to all. .
Velvet was replaced with:	Korean[7], English	The category, frequency, and length

Silk<br><br>Linen<br><br>Cotton<br><br>Teacup<br><br>Flannel<br><br>Nylon/Wool	(Singapore)/Chinese/Malay[10], Japanese[21], Filipino, Finnish, Hiligaynon[16], Indonesian[15]<br><br>Portuguese[22]<br><br>Sinhala[8], Arabic (Moroccan)[9]<br><br>Chinese-Language Los Angeles[20]<br><br>Chinese-Taiwan<br><br>Cantonese[12]	should be retained.<br><br>In Korean and Singaporean elderly "Velvet" is unfamiliar. "Silk was the replacement as it is of a similar frequency to ''velvet'' and helps reflect local familiarity.<br><br>In Filipino there is no translation for ''Velvet'', hence "Silk", a more commonly used fabric was chosen. There is also familiarity with "Silk".<br><br>In Finnish the word for "Silk" is shorter than the word for "Velvet" and it retains the number of syllables.<br><br>In Japanese "Velvet" is not commonly used. "Silk" shared the same frequency as "Velvet" in Japanese culture. The same holds true for Indonesian culture.<br><br>In Portuguese, ''Velvet'' was replaced with ''Linen'' because it is more recognisable to Portuguese adults.<br><br>In Sinhala people are unfamiliar with "Velvet". Similarly, Moroccan people are more familiar with "Cotton".<br><br>In Chinese "Velvet" can be either a 2-character or a 3- character word depending on the dialect and could be unfamiliar to older and low-educated Chinese. In contrast, "Teacup," is an unambiguous 2-character word in Chinese and familiar to all.<br><br>In Chinese different meanings can be represented by the same word. "Velvet" falls into this category. "Flannel" was chosen as the replacement as it maintains the 2 syllables as found in the English version.

		In Cantonese ‘‘Nylon’’ or ‘‘Wool’’ are unambiguous 2- character words and familiar to all.
Church was replaced with:<br><br>Temple<br><br>Mosque<br><br>Shrine<br><br>Shopping mall/Bank	Cantonese[6], Sinhala[8]<br><br>Iranian[23], Turkish[17–19], Urdu, Arabic (Moroccan)[9], Indonesian[15]<br><br>Japanese[21]<br><br>Cantonese[12]	The category, frequency, and length should be retained.<br><br>In Hong Kong, “Temple” is far more familiar to adults than “Church”.<br><br>In Sinhala the majority of people are Buddhist and are more familiar with the term ‘‘Temple’’.<br><br>In countries such as Iran, Turkey, Indonesia and Arab-speaking countries, where the predominant religion is Islam, people are more familiar with a ‘‘Mosque’’ than a ‘‘Church’’.<br><br>In Urdu, based on cultural and religious beliefs, the word for “Mosque” was more familiar with the elderly.<br><br>In Japan, ‘‘Shrine’’ is far more familiar to adults than ‘‘Church’’. “Shrine” also has the same frequency as “Church” in Japanese culture.<br><br>In Cantonese ‘‘Shopping mall’’ or ‘‘Bank’’ are unambiguous words that retain domain specificity and familiarity to all.
Daisy was replaced with:<br><br>Rose<br><br>Lily<br><br>‘‘Araliya’’ flower<br><br>Azalea<br><br>Tulip<br><br>Chrysanthemum<br><br>Lily of the Valley	English (Singapore)/Chinese/Malay[10], Filipino, Hiligaynon[16]<br><br>Japanese[21]<br><br>Sinhala[8]<br><br>Korean[7]<br><br>German[18]<br><br>Chinese-Taiwan<br><br>Finnish	The category, frequency, and length should be retained.<br><br>In Singapore, “Daisy” was replaced with “Rose” to help reflect local familiarity<br><br>In Filipino there is no translation for ‘‘Daisy’’, hence ‘‘Rose’’, a more commonly seen flower was the replacement.<br><br>In Japanese, taking the number of syllables, category and frequency of linguistic equivalents into consideration, ‘‘Daisy’’ was changed

Rhododendron/gladiolus<br><br>Hyacinth<br><br>Sunflower<br><br>Jasmine	Cantonese[12]<br><br>Serbian<br><br>Creole<br><br>Urdu, Arabic (Moroccan)[9]	into ''Lily''. "Daisy" was not well known in Japanese culture.<br><br>In Sinhala ''Daisy'' was replaced by the name of a common flower in Sri Lanka.<br><br>In Korean ''Daisy'' was changed to ''Azalea'' because ''Daisy'' is unfamiliar to Korean elderly. In addition, it is in the same category, has a similar frequency and is common.<br><br>In Chinese, meanings can be represented by the same word. "Daisy" falls into this category with it also being a type of imported flower. "Chrysanthemum" was the replacement as it maintains the 2 syllables as found in the English version.<br><br>In Finnish the word "Lily of the Valley" in is shorter than "Daisy" and retains the number of syllables.<br><br>In Cantonese ''Rhododendron'' or ''Gladiolus'' are unambiguous words that retain domain specificity and familiarity to all.<br><br>In Serbian "Daisy" translates to two words. The Serbian word for "Hyacinth" retains the number of syllables and is commonly found.<br><br>In Creole the word for "Daisy" did not achieve the conceptual, linguistic and semantic equivalence and was not culturally appropriate since it's typically also a woman's name. The word for "Sunflower" has intermediate frequency and more appropriate to the Cabo- Verdean reality.<br><br>In Urdu and Arabic, the word for "Daisy" is not very familiar and is not as well-known as the word for "Jasmine". This was also based on

		cultural and religious beliefs. Initially, it was to be replaced by the word for "Rose" but this was confused with the word for "Red".
Red was replaced with:<br><br>Blue<br><br>Purple/Violet<br><br>Black<br><br>Orange	Portuguese[22], Filipino, Creole, Hiligaynon[16]<br><br>Turkish[17–19], Cantonese[12]<br><br>Finnish<br><br>Cantonese[12]	The category, frequency, and length should be retained.<br><br>In Portuguese "Red" is a tri- syllabic word and the word itself in Portuguese sounds similar to "Marigold". Hence the colour ''Blue'' was the replacement, which has a similar number of syllables.<br><br>In Filipino due to the phonemic cue in translation of the word 'Red', 'Blue' was chosen as it still preserves the same thematic content and bi-syllabic translation.<br><br>In Creole the word for "Red" did not achieve the conceptual, linguistic and semantic equivalence and was not culturally appropriate. The word for "Blue" matched all the criteria.<br><br>In Turkish the word for ''Red'' is a 7 letter, multi-syllabic word, therefore ''Purple/ Violet'' was the replacement as it is a three-letter monosyllabic word.<br><br>In Finnish the word "Black" is shorter than "Red".<br><br>In Cantonese ''Purple'' or ''Orange'' are unambiguous words that retain domain specificity and familiarity to all.

For the Polish adaptation of the MoCA version 7.2, the original ''**truck – banana – violin – desk – green**'' were changed to ''**tap – pineapple – violin – table – white''**, respectively[13]. This was done based on using words that have an average occurrence in the local language, should share the same or similar number syllables to the original, should belong to similar semantic categories as in English and should phonetically sound the same as the original version[13].

QUESTION 6. ATTENTION: Digit Span

Read list of digits (1 digit/ sec.).	Subject has to repeat them in the forward order	[ ] 2 1 8 5 4
	Subject has to repeat them in the backward order	[ ] 7 4 2

Culturally adapted replacement	Language	Reason
Alternative digits used	Cantonese[12]	In Cantonese-Hong Kong versions alternative digits were chosen as they retained domain specificity and are relevant to the cultural and demographic background of the intended users.

QUESTION 7. ATTENTION: Vigilance

Read list of letters. The subject must tap with his hand at each letter A. No points if ≥ 2 errors

[ ] F B A C M N A A J K L B A F A K D E A A A J A M O F A A B

Instructions for this item were translated directly.

Culturally adapted replacement	Language	Reason
Arabic numerals were used instead of Roman alphabets	Chinese[1–5]<br><br>Singaporean[10]<br><br>Cantonese[11,12]	In Chinese speaking countries e.g. China, Singapore and Cantonese speaking countries e.g. Hong Kong, older adults struggle to be able to read the Roman alphabets, hence Arabic numerals are used- to help reflect local familiarity.<br><br>The numbers are in their relative positions to those of the alphabets. This creates a similar effect to the randomly placed alphabets
The Roman alphabets were replaced by Sinhala letters	Sinhala[8]	In Sinhala letters were chosen with a corresponding sound. In addition, this helps reflect local familiarity.
The Roman alphabets were replaced by Arabic letters	Arabic (Moroccan)[9]	In Arabic letters were chosen with a corresponding sound. In addition, this helps reflect local familiarity.

QUESTION 8. ATTENTION: Serial 7s

Serial 7 subtraction starting at 100

[ ] 93 [ ] 86 [ ] 79 [ ] 72 [ ] 65

Culturally adapted replacement	Language	Reason
Serial 7 subtraction starting at:<br>90<br>80	Cantonese[12]	In Cantonese-Hong Kong versions alternative start numbers were chosen for subtraction as they retained domain specificity and are relevant to the cultural and demographic background of the intended users

QUESTION 9. LANGUAGE: Sentence Repetition

Repeat : I only know that John is the one to help today. [ ]
The cat always hid under the couch when dogs were in the room. [ ]

Often, the item is directly translated however some cultural adaptation may be necessary.

Culturally adapted replacement	Language	Reason
**Sentence 1:**<br><br>The name ''John'' was replaced with a different name:<br><br>Nimal<br><br>Ali<br><br>Vano<br><br>Xiaowu<br><br>Juan<br><br>Jussi<br><br>Ji-su<br><br>Jon<br><br>Jan<br><br>Giovanni<br><br>Taro<br><br>João<br><br>Abdullah<br><br>Wati	Sinhala[8]<br><br>Iranian[23]<br><br>Georgian[24]<br><br>Chinese-Taiwan<br><br>Filipino<br><br>Finnish<br><br>Korean<br><br>Norwegian<br><br>Dutch<br><br>Italian<br><br>Japanese<br><br>Portuguese<br><br>Urdu<br><br>Indonesian[15]	In Sri Lanka the name ''Nimal'' is a common name.<br><br>In Iran ''Ali'' is a common name.<br><br>In Georgian ''Vano'' is the equivalent of ''John''. It is also a common name in Georgia and thus helps to reflect local familiarity.<br><br>In Taiwan ''John'' isn't a common name. ''Xiaowu'' was the replacement as it is a common last name randomly picked, that would give an impression just like "John" would give to one undergoing the test. "Xiao" means small or junior, while "Wu" is someone's last name. There are certain common last names among Chinese while people are used to calling others by their last name too.<br><br>In Filipino 'Juan' is the equivalent of 'John'. It also fits well with the sound of the translated sentence in Filipino.<br><br>In Creole, 'Juan' is the equivalent of 'John'.<br><br>In Finnish 'John' is an uncommon name in Finland, whereas 'Jussi' is a more common name.<br><br>In Korean ''Ji-su'' is the

		equivalent of ''John'' and is commonly used.<br><br>In Norwegian "Jon" is the equivalent of 'John' and regularly used in Norway. The name also consists of one syllable, not lengthening the sentence in the Norwegian translation.<br><br>In Dutch "John" is less frequently used in the Dutch language. "Jan" is the equivalent of "John", both names stemming from "Johannes", and "Jan" is frequently used in Dutch.<br><br>In Italian "Giovanni" is equivalent of "John".<br><br>In Japanese "Taro" is the equivalent of John and a typical name in Japan.<br><br>In Portuguese "João" is the equivalent of John.<br><br>In Urdu 'John' is unfamiliar to Pakistani elderly. The name 'Abdullah' was chosen because it's prevalent in the Pakistani culture and well known to the elderly population.<br><br>In Indonesia, "Wati" is the equivalent of John.
**Sentence 1 was changed to either:**<br><br>''Mom does yoga'' or<br><br>''Mom buys vegetables in wet market''	Cantonese[12]	In Cantonese-Hong Kong versions alternative sentences were chosen for sentence repetition as they retained domain specificity and are relevant to the cultural and demographic background of the intended users.
**Sentence 2:**<br><br>Translated to retain same number, however the meaning changed slightly. Back	Sinhala[8]	A meaningful sentence could be formed which has a similar meaning and the same number of words.

translation is as follows;<br><br>''when the dog barks the cat in our house runs and hides under the table''<br><br>**Sentence 2 was changed to either:**<br><br>''teacher teaches writing poems'' or<br><br>''Siu Ming doing high jumps on the playground''	Cantonese[12]	In Cantonese-Hong Kong versions alternative sentences were chosen for sentence repetition as they retained domain specificity and are relevant to the cultural and demographic background of the intended users.
The sentences in the Bahasa-Malaysia (BM) version were shortened:<br><br>Sentence 1: English 11-BM 9,<br><br>Sentence 2: English 13-BM 11<br><br>However, the number of syllables changed:<br><br>Sentence 1: English 13-BM 18,<br><br>Sentence 2: English 15-BM 25	Bahasa-Malaysia[25]	The sentences were shortened based on feedback from a pilot study.<br><br>This was done without altering the meaning or complexity of sentence structure.<br><br>Given the different syllable structure in BM and in English, it was not possible to match the sentences on both syllables and words.

QUESTION 10. LANGUAGE: Verbal Fluency

Fluency / Name maximum number of words in one minute that begin with the letter F

Often, the item is directly translated however some cultural adaptation may be necessary.

Culturally adapted replacement	Language	Reason
The phonemic category in the English version was replaced by semantic fluency task, involving the naming of different types of fruit/animals/ vegetables	Chinese[1–5]<br><br>Cantonese[6,11,12]<br><br>English (Singapore)/Chinese/Malay[10]<br><br>Chinese-Los Angeles[20]	In Chinese/Cantonese words there are no letters i.e. no letter-equivalent linguistic units. Words are not pronounced using phonemes but rather use a combination of consonants, vowels and tunes. Also, Chinese/ Cantonese-speaking older adults do not know English. Therefore, this was replaced by a semantic (category) fluency task, using the category of fruit/animals/ vegetables as this can also reflect one's executive function.<br><br>Chinese is a monosyllabic language and each phoneme has multiple ambiguous meanings, which undermines the meaningfulness of phonemic fluency in Chinese.
The phonemic category in the English version was replaced by semantic fluency task requiring the production of as many objects which could be bought in a market as possible.	Korean[7]	In Korean one character is one syllable which consists of several phonemes (letters). In addition, a fluency test beginning with a letter is unfamiliar to Korean elderly, and there are too few words beginning with a character to test fluency (language characteristics). Therefore, we changed the phonemic fluency test to the semantic fluency test.
The letter ''F'' was replaced by a different letter:<br><br>S<br><br>K	Sinhala[8], Finnish, Indonesian[15]<br><br>Georgian[24], German[26], Turkish[18]<br><br>German[26], Arabic (Lebanese)[27]	In Sinhala ''F'' is not used commonly with very few Sinhala words starting with that letter. ''S'' is more commonly used. The same holds true for Indonesian.

M<br><br>B<br><br>**R** (for version 7.2) and **T** (for version 7.3)<br><br>**S** (for version 7.2) and **P** (for version 7.3)<br><br>D<br><br>X<br><br>P<br><br>C<br><br>A<br><br>Ka<br><br>'Jeem' (J)<br><br>'Ba' (B)	Filipino, Hiligaynon[16]<br><br>**French**[14]<br><br>**Czech**[28]<br><br>Dutch<br><br>Greek<br><br>Portuguese<br><br>Serbian<br><br>Malay (Bahasa Malaysia)<br><br>Japanese<br><br>Urdu<br><br>Arabic (Moroccan)[9]	In Finnish "F" is not used. "S" is common in the Finnish language.<br><br>In Croatian words starting with the letter "S" are far more frequent than those starting with "F".<br><br>In Georgian and Turkish the number of words beginning with ''K'' is equivalent to the number of words beginning with ''F'' in English.<br><br>In German the letters "K" and "M" were the replacement to assure a similar letter frequency and to prevent possible differences in the task difficulty level between the alternate forms. Additionally, for the Arabic-Lebanese version of the MoCA, the letter 'F' was replaced with 'M' as the use of 'F' was problematic for the Lebanese population.<br><br>In Filipino and Hiligaynon there are no words beginning with "F" apart from the word Filipino itself. "B" was the replacement as there are more words starting with this letter. Also, compared to other Filipino/Hiligaynon consonants "B" is not used as a common prefix to different Filipino/Hiligaynon words e.g. action words, therefore they are good as a starting letter for the fluency test.<br><br>In Dutch the letter "D" has the same fluency as the letter "F" does in English.<br><br>In Greek the letter "X" is commonly used in fluency tasks within clinical practice.

		In Portuguese "P" has a similar lexical frequency as "F" in English, and it is commonly used in fluency tests in Portugal.<br><br>In Serbian words beginning with "F" are not as frequent as in English so a more frequent letter "C" was used.<br><br>In Malay, words beginning with "F" are not as frequency as in English. "A" is associated with a reasonable number of nouns.<br><br>In Japanese the character for Ka is commonly used in word fluency tasks.<br><br>In French the letters ''R'' and ''T'' were chosen as they are equally frequent letters found in the dictionary.<br><br>In Czech, the letters ''S'' and ''P'' were chosen as they are equally frequent letters found in the dictionary.<br><br>In Urdu fluency was calculated according to the number of words starting with the letter in the dictionary. The letter 'Jeem' which makes a "J" sound was found to have the same fluency as "F". Words beginning with 'Jeem' were also familiar with the elderly.<br><br>The letter 'Ba' in Arabic which makes a "B" sound was found to have the same fluency as "F". Words beginning with 'Ba' were also familiar with the elderly

QUESTION 11. ABSTRACTION

Similarity between e.g. banana - orange = fruit [ ] train – bicycle [ ] watch - ruler

Often, the item is directly translated however some cultural adaptation may be necessary.

Culturally adapted replacement	Language	Reason
''Watch'' was replaced by "Scale'', in the word pair ''Watch-Ruler''	Cantonese[6]	In Cantonese the pair "Watch–Ruler" was unfamiliar among Chinese older adults in Hong Kong.
One easier task and one harder task	French[14]	In French the same level of difficulty and frequency of the elements was respected.
**''Train-bicycle'' replaced by:**<br>''Harmonica-Guitar''/ ''Fork-Chopsticks''<br>**Watch-ruler replaced by:**<br>''Calligraphy-Painting''/ ''Pencil-Paper clips''	Cantonese[12]	In Cantonese-Hong Kong versions alternative word pairs were chosen for abstraction as they retained domain specificity and are relevant to the cultural and demographic background of the intended users.

QUESTION 12. MEMORY – Delayed Recall

Has to recall words **WITH NO CUE**	FACE [ ]	VELVET [ ]	CHURCH [ ]	DAISY [ ]	RED [ ]	Points for UNCUED recall only
Category cue						
Multiple choice cue						

This item is often directly translated; however, some cultural adaptation may take place.

Refer to Item 5. MEMORY: Recall

QUESTION 13. ATTENTION: Orientation

[ ] Date [ ] Month [ ] Year [ ] Day [ ] Place [ ] City

Often, the item is directly translated however some cultural adaptation may be necessary

Culturally adapted replacement	Language	Reason
"City" was replaced by "District".	Chinese[4]<br><br>Cantonese[6]	In Taiwan and Hong Kong, the districts there are the equivalent to cities in North America and Europe.

REFERENCES

1. Chang YT, Chang CC, Lin HS, et al. Montreal cognitive assessment in assessing clinical severity and white matter hyperintensity in Alzheimer's disease with normal control comparison. *Acta Neurol. Taiwan.* 2012;21(2):64–73. Available at: http://www.ant-tnsjournal.com/Mag_Files/21-2/004.pdf.

2. Hu JB, Zhou WH, Hu SH, et al. Cross-cultural difference and validation of the Chinese version of Montreal Cognitive Assessment in older adults residing in Eastern China: Preliminary findings. *Arch. Gerontol. Geriatr.* 2013;56(1):38–43. Available at: http://ovidsp.ovid.com/ovidweb.cgi?T=JS&PAGE=reference&D=emed15&NEWS=N&AN=52056801.

3. Nie K, Zhang Y, Wang L, et al. A pilot study of psychometric properties of the Beijing version of Montreal Cognitive Assessment in patients with idiopathic Parkinson's disease in China. *J. Clin. Neurosci.* 2012;19(11):1497–1500. Available at: http://ovidsp.ovid.com/ovidweb.cgi?T=JS&PAGE=reference&D=emed14&NEWS=N&AN=52195163.

4. Tsai CF, Lee WJ, Wang SJ, Shia BC, Nasreddine Z, Fuh JL. Psychometrics of the Montreal Cognitive Assessment (MoCA) and its subscales: Validation of the Taiwanese version of the MoCA and an item response theory analysis. *Int. Psychogeriatrics* 2012;24(4):651–658. Available at: http://ovidsp.ovid.com/ovidweb.cgi?T=JS&PAGE=reference&D=emed14&NEWS=N&AN=364517082.

5. Yu J, Li J, Huang X. The Beijing version of the montreal cognitive assessment as a brief screening tool for mild cognitive impairment: A community-based study. *BMC Psychiatry* 2012;12(1):156. Available at: http://www.biomedcentral.com/content/pdf/1471-244X-12-156.pdf.

6. Chu LW, Ng KHY, Law ACK, Lee AM, Kwan F. Validity of the Cantonese Chinese Montreal cognitive assessment in Southern Chinese. *Geriatr. Gerontol. Int.* 2015;15(1):96–103. Available at: http://www.blackwellpublishing.com/journals/ggi.

7. Lee JY, Dong Woo L, Cho SJ, et al. Brief screening for mild cognitive impairment in elderly outpatient clinic: Validation of the Korean version of the Montreal Cognitive Assessment. *J. Geriatr. Psychiatry Neurol.* 2008;21(2):104–110.

8. Karunaratne S, Hanwella R, de Silva V. Validation of the Sinhala version of the Montreal Cognitive Assessment in screening for dementia. *Ceylon Med. J.* 2011;56(4):147–153. Available at: http://www.ncbi.nlm.nih.gov/pubmed/22298207. Accessed February 14, 2020.

9. Benabdeljlil M, Azdad A, Mustapha EAF. Standardization and validation of montreal cognitive assessment (MoCA) in the moroccan population. *J. Neurol. Sci.* 2017;381:318. Available at: http://article.sapub.org/10.5923.j.ijbcs.20190801.01.html#Sec1. Accessed July 18, 2020.

10. Dong Y, Sharma VK, Chan BPL, et al. The Montreal Cognitive Assessment (MoCA) is superior to the Mini-Mental State Examination (MMSE) for the detection of vascular cognitive impairment after acute stroke. *J. Neurol. Sci.* 2010;299(1–2):15–18. Available at: http://ovidsp.ovid.com/ovidweb.cgi?T=JS&PAGE=reference&D=emed12&NEWS=N&AN=51095450.

11. Wong A, Xiong YY, Kwan PWL, et al. The validity, reliability and clinical utility of the Hong Kong

Montreal Cognitive Assessment (HK-MoCA) in patients with cerebral small vessel disease. *Dement. Geriatr. Cogn. Disord.* 2009;28(1):81–87. Available at: http://ovidsp.ovid.com/ovidweb.cgi?T=JS&PAGE=reference&D=emed12&NEWS=N&AN=50610398.

12. Wong A, Yiu S, Nasreddine Z, et al. Validity and reliability of two alternate versions of the Montreal Cognitive Assessment (Hong Kong version) for screening of Mild Neurocognitive Disorder. Brucki S, ed. *PLoS One* 2018;13(5):e0196344. Available at: http://dx.plos.org/10.1371/journal.pone.0196344. Accessed June 24, 2019.

13. Gierus J, Mosiolek A, Koweszko T, et al. The montreal cognitive assessment 7.2 - Polish adaptation and research on equivalency. *Psychiatr. Pol.* 2015;49(1):171–179. Available at: http://www.psychiatriapolska.pl/uploads/images/PP_1_2015/171Gierus_PsychiatrPol2015v49i1.pdf.

14. Nasreddine ZS, Patel BB. Validation of Montreal Cognitive Assessment, MoCA, Alternate French Versions. *Can. J. Neurol. Sci.* 2016;43(5):665–671. Available at: http://journals.cambridge.org/action/displayBackIssues?jid=CJN.

15. Husein N, Lumempouw S, Ramli Y, Herqutanto. Uji validitas dan reliabilitas Montreal Cognitive Assesment versi Indonesia (MoCA-Ina) untuk skrining gangguan fungsi kognitif. *Neurona* 2010;27(4):15–22. Available at: http://www.neurona.web.id/paper-detail.do?id=734. Accessed July 18, 2020.

16. Aliling NB, Rivera AS, Jamora RDG. Translation, Cultural Adaptation, and Validation of the Hiligaynon Montreal Cognitive Assessment Tool (MoCA-Hil) Among Patients With X-Linked Dystonia Parkinsonism (XDP). *Front. Neurol.* 2019;10. Available at: /pmc/articles/PMC6892978/?report=abstract. Accessed July 18, 2020.

17. Ozdilek B, Kenangil G. Validation of the Turkish Version of the Montreal Cognitive Assessment Scale (MoCA-TR) in patients with Parkinson's disease. *Clin. Neuropsychol.* 2014;28(2):333–343.

18. Krist L, Keller T, Sebald L, et al. The Montreal Cognitive Assessment (MoCA) in a population-based sample of Turkish migrants living in Germany. *Aging Ment. Health* 2019;23(1):30–37. Available at: https://www.tandfonline.com/doi/full/10.1080/13607863.2017.1396577. Accessed June 24, 2019.

19. Selekler K, Cangöz B, Uluç S. Power of discrimination of Montreal Cognitive Assessment (MOCA) scale in Turkish patients with mild cognitive impairement and Alzheimer's disease. *Turk Geriatr. Derg.* 2010;13(3):166–171. Available at: www.mocatest.org. Accessed July 18, 2020.

20. Zheng L, Teng EL, Varma R, et al. Chinese-language montreal cognitive assessment for cantonese or mandarin speakers: Age, education, and gender effects. *Int. J. Alzheimers. Dis.* 2012:204623. Available at: http://ovidsp.ovid.com/ovidweb.cgi?T=JS&PAGE=reference&D=emed14&NEWS=N&AN=365424883.

21. Fujiwara Y, Suzuki H, Yasunaga M, et al. Brief screening tool for mild cognitive impairment in older Japanese: Validation of the Japanese version of the Montreal Cognitive Assessment. *Geriatr. Gerontol. Int.* 2010;10(3):225–232. Available at: http://www.blackwellpublishing.com/journals/ggi.

22. Freitas S, Simões MR, Martins C, Vilar M, Santana I. Estudos de adaptação do Montreal

Cognitive Assessment (MOCA) para a população portuguesa. *Avaliação Psicológica* 2010;9(3):345–357.

23. Emsaki G, Molavi H, Chitsaz A, Abtahi MM, Asgari K. Psychometric properties of the montreal cognitive assessment (MoCA) in Parkinson's disease patients in Isfahan. *J. Isfahan Med. Sch.* 2011;29(158). Available at: http://jims.mui.ac.ir/index.php/jims/issue/archive.

24. Janelidze M, Mikeladze N, Bochorishvili N, et al. Validity of the Georgian Montreal Cognitive Assessment for the Screening of Mild Cognitive Impairment and Dementia. *Am. J. Alzheimers. Dis. Other Demen.* 2017;32(1):36–40. Available at: http://aja.sagepub.com/archive/.

25. Sahathevan R, Mohd Ali K, Ellery F, et al. A Bahasa Malaysia version of the Montreal Cognitive Assessment: Validation in stroke. *Int. Psychogeriatrics* 2014;26(5):781–786. Available at: http://uk.cambridge.org/journals/journal_catalogue.asp?historylinks=ALPHA&mnemonic=IPG.

26. Costa AS, Fimm B, Friesen P, et al. Alternate-form reliability of the montreal cognitive assessment screening test in a clinical setting. *Dement. Geriatr. Cogn. Disord.* 2012;33(6):379–384.

27. Hayek M, Tarabey L, Abou-Mrad T, Fadel P, Abou-Mrad F. Normative Data for the Montreal Cognitive Assessment in a Lebanese Older Adult Population. *J. Clin. Neurosci.* 2020;74:81–86.

28. Bezdíček O, Georgi H, Panenková E, et al. Equivalence of montreal cognitive assessment alternate forms. *Ces. a Slov. Neurol. a Neurochir.* 2019;82(3):332–340.
