# Supplementary material for: Developing guidelines for the translation and cultural adaptation of the Montreal Cognitive Assessment: scoping review and qualitative synthesis
Source: BJPsych Open. 2022 Jan 7;8(1):e21. doi: 10.1192/bjo.2021.1067 (PMC8811786; doi:10.1192/bjo.2021.1067)
Supplement: Supplementary file 1 [file bjosup.zip › S205647242101067Xsup003.docx]

**Full-text articles that could not be located**

1. Badrkhahan SZ, Sikaroodi H, SharifiF, KoutiL, Noroozian M. Validityand reliability of the Persian version of the Montreal Cognitive Assessment (MoCA-P) scale among subjects with Parkinson's disease. Appl Neuropsychol Adult 2020; 27: 431–9.
2. Bartos A, Fayette D. Validation of the Czech Montreal Cognitive Assessment for mild cognitive impairment due to Alzheimer disease and Czech norms in 1,552 elderly persons. Dement Geriatr Cogn Disord 2018; 46: 335–45.
3. Bleecke M, de Jongue J, Oremus M, Boelaarts M. Psychometric Properties of the Montreal Cognitive Assessment Dutch Version (MoCA-D). A Comparison with the MMSE in Mild Cognitive Impaired (MCI) Patients. Master‘s thesis. 2006.
4. Duro D. Montreal Cognitive Assessment (MoCA): psychometric propertiesand diagnostic capacity in a memory consult setting. Master's thesis. 2008.
5. Huang Y-Y, Qian S-X, Guan Q-B, Chen K-L, Zhao Q-H, Lu J-H, et al. Comparative study of two Chinese versions of Montreal Cognitive Assessment for screening of mild cognitive impairment. Appl Neuropsychol Adult 2021; 28: 88–93.
6. Thomas PE, Sheetal Raj M, Adhikari P, Kotian S, Raj MS, Adhikari P, et al. Validityand reliability of Montreal Cognitive Assessment and its comparison with the translated Hindi Mental StatusExamination in a South Indian geriatric population. Indian J Public Health Res Dev 2018; 9: 173–7.
