# Supplementary material for: Developing guidelines for the translation and cultural adaptation of the Montreal Cognitive Assessment: scoping review and qualitative synthesis
Source: BJPsych Open. 2022 Jan 7;8(1):e21. doi: 10.1192/bjo.2021.1067 (PMC8811786; doi:10.1192/bjo.2021.1067)
Supplement: Supplementary file 1 [file bjosup.zip › S205647242101067Xsup002.doc]

**CULTURAL ADAPTATION PROCESS OF THE FILIPINO MOCA**

You have been asked to complete the following questionnaire which will ask you to elaborate on the adaptation process you undertook when developing the Filipino Montreal Cognitive Assessment. Questions will refer to items that were changed. Upon completion please return to ghazn.khan@student.manchester.ac.uk

**Q1: Was the Filipino MOCA mentioned in any publications? Please list.**

Answer:

**Q2a: In the “Naming” item, what was the justification for changing the animal?**

ORIGINAL:

ENGLISH (SINGAPORE):

Answer:

**Q2b: How did you select the animal that would take its place? What was the rationale behind this selection?**

Answer:

**Q3a: In the “Memory” item, what was the justification for changing the following words?**

ORIGINAL:

RED

VELVET

DAISY

FILIPINO:

ASUL (BLUE)

SEDA (SILK)

ROSAS (ROSE)

Answer:

**Q3b: How did you select the words that would take their place? What was the rationale behind this selection?**

Answer:

**Q4a: In the first “Language” item, what was the justification for changing the name John?**

Answer:

**Q4b: How did you select ‘Juan’ to take its place? What was the rationale behind this choice?**

Answer:

**Q5a: In the second “Language” item, what was the justification for changing the letter ‘F’?**

Answer:

**Q5b: How did you select ‘B’ to take its place? What was the rationale behind this selection?**

Answer:

**Thank you for taking the time to complete this questionnaire.**
